# Pyronaridine exerts potent cytotoxicity on human breast and hematological cancer cells through induction of apoptosis

**DOI:** 10.1371/journal.pone.0206467

**Published:** 2018-11-05

**Authors:** Paulina J. Villanueva, Alberto Martinez, Sarah T. Baca, Rebecca E. DeJesus, Manuel Larragoity, Lisett Contreras, Denisse A. Gutierrez, Armando Varela-Ramirez, Renato J. Aguilera

**Affiliations:** 1 The Cytometry, Screening and Imaging Core Facility & Border Biomedical Research Center & Department of Biological Sciences, the University of Texas at El Paso, El Paso, Texas, United States of America; 2 Chemistry Department, New York City College of Technology, The City University of New York, Brooklyn, New York, United States of America; University of South Alabama, UNITED STATES

## Abstract

The potent antimalarial drug pyronaridine (PND) was tested for its potential as an anticancer drug. After exposing cancerous (17) and non-cancerous (2) cells to PND for 72 hr, PND was found to exhibit consistent and potent cytotoxic activity at low micromolar (μM) concentrations that ranged from 1.6 μM to 9.4 μM. Moreover, PND exerted a significant selective cytotoxicity index (SCI) on five out of seven breast cancer cell lines tested, with favorable values of 2.5 to 4.4, as compared with the non-cancerous breast MCF-10A cell line. By using the same comparison, PND exhibited a significant SCI on three out of four leukemia/lymphoma cell lines with promising values of 3.3 to 3.5. One breast cancer and one leukemia cell line were tested further in order to determine the likely mode of action of PND. PND was found to consistently elicit phosphatidylserine externalization, mitochondrial depolarization, and DNA fragmentation, in both the triple negative MDA-MB-231 breast cancer and HL-60 leukemia cell lines. In addition, PND treatment altered cell cycle progression in both cancer cells. Subsequent DNA mobility-shift assays, UV-Visible spectroscopic titrations, and circular dichroism (CD) experiments revealed that PND intercalates with DNA. The findings presented in this study indicates that PND induces apoptosis and interfered with cell cycle progression of cancer cell lines and these results indicate that this drug has the potential as a repurposed drug for cancer therapy.

## Introduction

The drug pyronaridine (PND) is a benzonaphthyridine derivative initially synthesized in 1970 at the Institute of Chinese Parasitic Disease and has been used in China for over 30 years for the treatment of malaria [1]. Previous reports indicated that PND inhibits β-hematin formation promoting β-hematin-induced red blood cell lysis based on *in vitro* studies of *Plasmodium falciparum* K1 [2]. It was also suggested that PND could be an inhibitor of DNA topoisomerase II of *P*. *falciparum* provoking the formation of a PND-DNA topoisomerase II-DNA complex [3]. Although PND did not generate the formation of protein-DNA complexes, PND did inhibit *P*. *falciparum* parasitic DNA topoisomerase II activity *in vitro* [1]. In addition, PND was tested alone and in combination with doxorubicin (DOX) on multidrug-resistant (MDR) K562/A02 and MCF-7/ADR human cancer cells and found to increase the sensitivity of cells to doxorubicin [4]. The growth inhibitory effects of PND were tested on several cancer cell lines but the mechanism of action was not determined in this or in earlier work from the same group [4,5]. The growth inhibitory effects of PND were tested on several cancer cell lines but the mechanism of action was not determined in this or in earlier work from the same group [4,5]. PND was also found to exhibit the same DOX sensitizing effect in mice carrying the same human MDR tumor xenografts (K562/A02 and MCF-7/ADR cells) and did not exhibit toxicity to treated mice [4]. In a recent report, it was demonstrated that nanorods containing both PND and DOX could efficiently kill MDR MCF-7/ADR cells [6]. However, in this most recent report, PND was only administered in combination with DOX and therefore it could not be determined if PND had an effect by itself [6]. Since the mechanism by which PND exerts cell death was not previously determined, we sought to determine if PND induces apoptosis using a variety of *in vitro* assays and have shown that PND intercalates with DNA and negatively affects cell cycle progression.

In this study, we determined that PND is able to induce effective cytotoxicity as a single agent on human breast and hematological cancer cells, and exhibits a favorable selective cytotoxicity index (SCI), as compared with non-cancerous cells. Furthermore, PND was found to induce apoptosis *via* mitochondrial depolarization, Caspase 3 activation, inhibition of cell cycle progression and by directly intercalating with cellular DNA. Since it has been shown that PND is relatively safe to use in humans suffering from malaria, it could also have potential use as a human therapeutic against cancer.

## Materials and methods

### Preparation of pyronaridine tetraphosphate-PND

**Fig 1:**
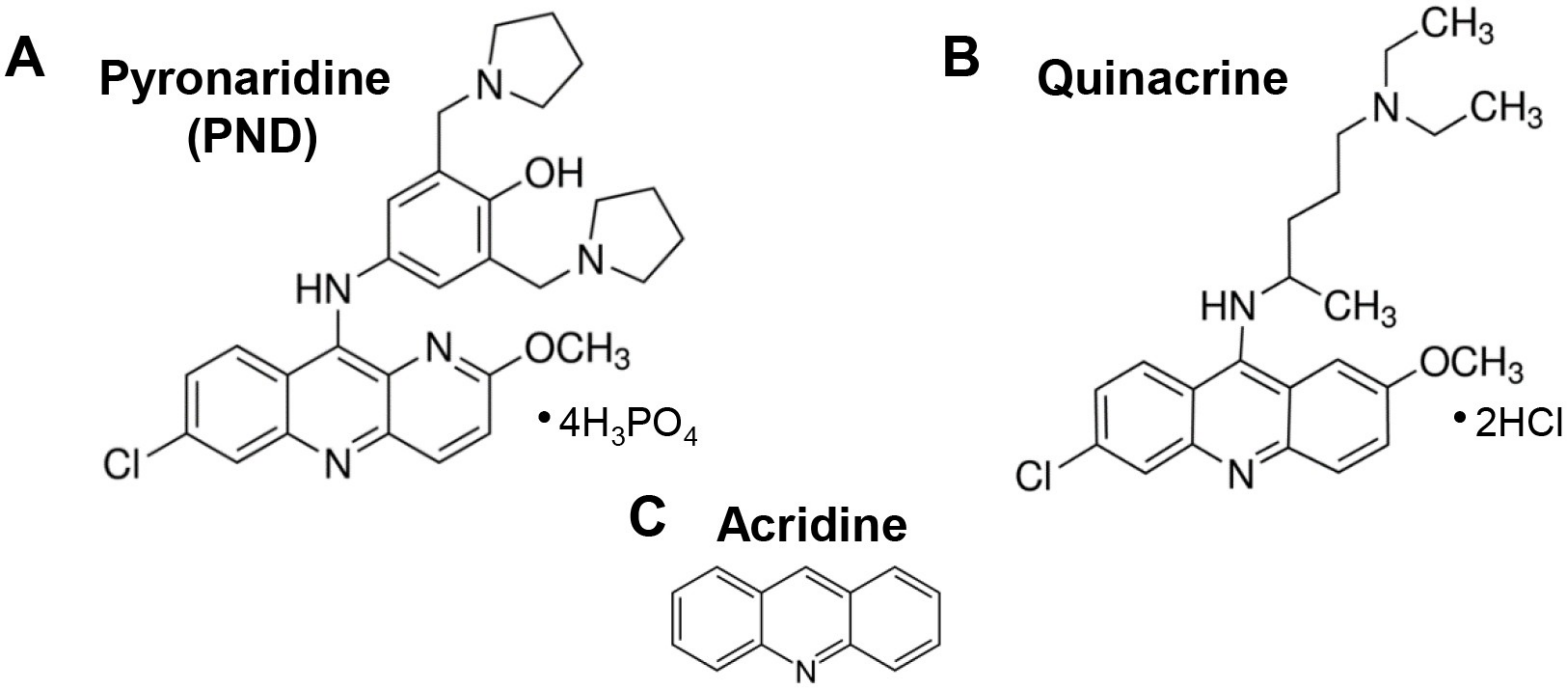
The chemical structures of pyronaridine tetraphosphate (A), quinacrine dihydrochloride (B) and acridine (C) are depicted for comparative purposes. Quinacrine and acridine are related antimalarial compounds with similar chemical structures to PND and used as controls in some experiments.

Pyronaridine tetraphosphate (PND; 2-methoxy-7-chloro-10[3,5-bis(pyrrolidinyl-1-methyl-)4hydroxyphenyl]aminobenzyl-(b)-1,5-naphthyridine; APExBIO, Houston, TX, USA) stock solution and PND diultions were freshly prepared by using Dulbecco’s Phosphate Buffered Saline (PBS; Sigma-Aldrich, St Louis, MO, USA) as a solvent. Both the sock solutions and their dilutions were added directly to the wells containing cells in culture media. The chemical structures of pyronaridine tetraphosphate, quinacrine dihydrochloride and acridine are depicted for comparative purposes (Fig 1A, 1B and 1C, respectively).

### Cell lines and culture conditions

In this study, 19 human cell lines were utilized: seven breast human cancer; MDA-MB-468, MCF-7, T47D, HCC1419, HCC70, MDA-MB-231 (triple negative) and its lung metastatic (LM) derivative MDA-MB-231 LM2; four human leukemia/lymphoma cells, HL-60, Ramos, Jurkat and CEM,three human ovarian cancer, Ovcar 8, Ovcar 5 and Ovcar 3, one lung cancer, one melanoma and one pancreatic cell line, A549, A375, Panc-1 respectively. Furthermore, for selectivity/comparative purposes, cell lines from the non-cancerous origin, MCF-10A, HS-27 were also included. The culture media used for MDA-MB-231, MDA-MB-231 LM2, MDA-MB-468, MCF-7, A549, A375, Panc-1, and HS-27 was DMEM (Hyclone, Logan UT), whereas for T47D, HCC-1419, HCC-70, HL-60, Ramos, Jurkat, CEM, Ovcar 8 and Ovcar 5 was RPMI-1640 (Hyclone, Logan UT). Consistently, both DMEM and RPMI culture media was supplemented with 10% heat-inactivated fetal bovine serum (FBS: Hyclone), 100 U/ml penicillin and 100 μg/ml streptomycin (Thermo Fisher Scientific Inc., Rockford, IL). The MCF-10A cells were grown in DMEM/F12 supplemented with 10% FBS, 10 μg/ml recombinant human insulin (Sigma), 0.5 μg/ml hydrocortisone (Sigma), 20 ng/ml epidermal growth factor, 2.5 mM L-glutamine, 100 U/ml penicillin, and 100 μg/ml streptomycin. Also, a slight modification of the culture media to the HL-60 and Ovcar 3 cell lines was that 20% of FBS was added to the media (as recommended by ATCC, Manassas, VA, USA). In addition, Ovcar 3 cells required 10 μg/ml recombinant human insulin (Sigma). The adherent cells, growing in the logarithmic growth phase at a 60–75% confluence, were detached by utilizing a HyQtase enzyme (Thermo Fisher Scientific Inc.), counted and seeded in 96-well plates at 10,000 cells density in 100 μL of culture medium per well. Cells growing in suspension were processed similarly as described above except for the addition of HyQtase. Typically, the incubation conditions of all the cells were at 37°C in a 5% CO_2_ humidified atmosphere.

### Differential nuclear staining assay to quantify cell death

To analyze the potential cytotoxic activity of PND, the Differential Nuclear Staining (DNS) assay, which was validated for high-throughput screening (HTS) using live-cell bio-imaging was utilized [7]. For this assay, cells were seeded at 10,000 cells/well density in a 96-well plate in 100 μl of culture media, incubated overnight, and treated with a gradient of PND concentrations for 72 h. Two hours before imaging, two fluorescent nucleic acid intercalators were added to each well, Hoechst 33342 and Propidium iodide (PI; Invitrogen); at a final concentration of 1 μg/ml each. Due to its high permeability, Hoechst stains all of the cells (total dead and alive), whereas PI only stains dead or dying cells. Montages of 2 by 2 images were captured directly from each individual well of the culture plates by using a multi-well plate reader IN Cell 2000 analyzer, an HTS and high-content analysis (HCA) system (GE Healthcare Life Sciences, Pittsburgh, PA). The following controls were included in every single plate: PBS as solvent/vehicle control, Hydrogen peroxide as a positive control for cytotoxicity, and untreated cells to determine the background of toxicity due to cell manipulation and intrinsic factors usually associated with the culture protocol. Each experimental data point, as well as controls, were assessed in triplicates. Cytotoxic concentration 50% (CC_50_) values were calculated based on a linear interpolation equation as previously described [8]. CC_50_ is defined as the PND concentration required to disrupt the plasma membrane integrity of 50% of the cell population, as compared with solvent-treated cells [9].

### Selective cytotoxicity index calculation

The selective cytotoxicity index (SCI) denotes the capability of a given experimental compound to kill cancer cells more efficiently while inflicting minimal toxicity to non-cancerous origin cells. Thus, the SCI for PND was calculated as follow: SCI = CC_50_ of non-cancerous cells / CC_50_ of cancer cells, as described previously [10].

### Analysis of phosphatidylserine externalization via annexin V/PI assay

Phosphatidylserine (PS) externalization is an early feature of apoptosis induction that can be readily detected with the high-affinity PS ligand annexin V–FITC *via* flow cytometry [10,11,12]. Cells were seeded in 24-well plates at a density of 100,000 for adherent MDA-MB-231 and 200,000 HL-60 cells in 1 ml of culture media. After overnight incubation, cells were treated with PND, and incubated for an additional 24 h. For MDA-MB-231 cells, unattached cells were harvested in an ice-cold tube, while adhered cells were detached by using HyQtase (Thermo Fisher) and incubated for 5 min at 37 °C. Both unattached and detached cells harvested from each individual well were washed with ice-cold PBS and centrifuged at 260 xg for 5 min. HL-60 cells were centrifuged directly after the incubation period as they grow in suspension. Cells were then stained with a mixture of annexin V-FITC and PI in 100 μl of binding buffer and incubated on ice in the dark for 15 minutes, following the manufacturer’s instructions (Beckman Coulter). Lastly, the cells were resuspended in 400 μl of ice-cold binding buffer and analyzed by flow cytometry (Cytomics FC500; Beckman Coulter). For this series of experiments, cells treated with PBS, as solvent control; treated with H_2_O_2_, as a positive control of cytotoxicity; and untreated were included and processed in parallel. For each sample, 10,000 events/cells were collected and analyzed using CXP software (Beckman Coulter). Both the experimental samples and their controls were processed similarly and assessed in triplicate. The sum of both early and late stages of apoptosis was calculated to obtain the total percentage of apoptotic cells.

### Polychromatic analysis of mitochondrial membrane potential

MDA-MB-231 and HL-60 cells were seeded as described in the previous section and treated with PND for 7 h. After treatment, the cells were harvested and stained with 2 μM of the fluorophore 5,5',6,6'-tetrachloro-1,1',3,3'- tetraethylbenzimidazolylcarbocyanine iodide (JC-1) following manufacturer’s instructions (MitoProbe; Life Technologies, Grand Island, NY, USA). Cells with healthy polarized mitochondria favor for JC-1 to form aggregates, which emit a red signal. Cells that have a depolarized mitochondria exhibit a green signal, due to the dispersed JC-1 monomers. Similar controls as above were concurrently analyzed. Data acquisition and analysis were achieved by using CXP software (Beckman Coulter). Each data point was analyzed in triplicate.

### Analysis of the transitions between cell cycle phases

Asynchronous cultures in the exponential growth phase of MDA-MB-231 and HL-60 cells in 24-well plates were treated with several doses of PND. After 72 h of incubation, cells were centrifuged and treated as in the previous section, fixed, permeabilized and stained with a DNA intercalating fluorophore, 4,6-Diamidino-2-phenylindole (DAPI); those three steps were accomplished by adding to the cells 200 μl of a single nuclear isolation medium (NIM)-DAPI solution (Beckman Coulter) [13]. The cell suspension was then incubated for an additional 3 min at room temperature in the dark [10]. The controls included in the series of experiments were as described for the previous experiments. Approximately, 20,000 events/cells were collected *per* sample by using a flow cytometer equipped with a solid state 405 nm laser (Gallios; Beckman Coulter). The acquisition and distribution of cell subpopulations within of each cell cycle facets were accomplished by utilizing Kaluza flow cytometry software (Beckman Coulter). Additionally, doublets were effectively eliminated by including a single cells gate in the acquisition cell cycle protocol.

### DNA mobility-shift assay

Typically, an experimental compound intercalating or binding to double stranded (ds) DNA will increase the molecular mass after forming complexes that decrease its electrophoretic mobility in an agarose gel, as compared with untreated DNA. To explore the potential interaction between PND and DNA, a DNA mobility-shift assay was conducted. Each reaction mixture was of 10 μl total volume in PBS pH 7.4. Both PND and quinacrine (Sigma-Aldrich) were tested at three individual concentration of 1 μM, 0.5 μM or 0.25 μM, respectively. To each reaction mixture, 100 ng of plasmid double-stranded (ds) DNA (pCMV-dR8.91; Addgene, Cambridge, MA) was added and the mixture was then incubated for 30 min at 37°C and stopped by adding 2 μl of 6X gel loading buffer and placed on ice. The potential binding interaction between both PND and quinacrine with dsDNA were analyzed by using 1% (w/v)-agarose-gel electrophoresis dissolved in TAE buffer (0.04 M Tris base, 0.04 M acetate and 0.001 M EDTA) pH 8.0. To stain the dsDNA complexes, Ethidium bromide was added to the agarose gel throughout the electrophoresis process at a concentration of 0.5 μg/ml. DNA migration was visualized by utilizing a gel documentation system and pictures were captured by using a UV-light trans-illuminator (Alpha Innotech, San Leandro, CA) [14]. The well-known DNA intercalator fluorescent compound, Propidium iodide, was included as a positive control at 1 μg per reaction and untreated dsDNA was included to determine its normal electrophoretic mobility in the gel.

### Analysis of the interaction of PND with Calf Thymus DNA by UV-Visible spectroscopy

UV-Visible measurements were taken in a Varian Cary 100 spectrophotometer. *Calf Thymus* (CT) DNA and buffers were purchased from Sigma Aldrich. The binding constant of PND with CT DNA was determined by absorption titration at room temperature through stepwise addition of a CT DNA solution (10.1 mM; 5 μL additions) in buffer (5 mM Tris/HCl, 50 mM NaCl, pH = 7.39) over a 2 mL working solution of PND (22.8 μM) in the same buffer. Absorption spectra were recorded at 424 nm and the titration was terminated when saturation was reached. In order to determine the binding affinity, the data was fitted to the Scatchard equation r/C_f_ = K(n-r) (McGhee and von Hippel plots) [15,16], where r is the number of moles of PND bound to 1 mol of CT DNA, n is the number of equivalent binding sites, and K is the affinity of the complex for those binding sites. Concentrations of free (C_f_) and bound (C_b_) complexes were calculated from C_f_ = C(1−α) and C_b_ = C−C_f_, respectively, where C is the total PND concentration. The fraction of the bound complex (α) was calculated from α = (A_f_−A)/(A_f_−A_b_), where A_f_ and A_b_ are the absorbances of the free and fully bound drug at the selected wavelengths, and A is the absorbance at any given point during the titration. The plot of r/C_f_ vs. r gives the binding constant K_b_ as the slope of the graph [17]. All experiments were performed in triplicate and values of K_b_ were averaged.

### Analysis of the interaction of PND with Calf Thymus DNA via circular dichroism spectroscopy

Circular dichroism (CD) spectra measurements were taken in a JASCO-1100 spectropolarimeter equipped with a Xenon lamp (JASCO, Easton, MD). A CT DNA stock solution was prepared in Tris/HCl buffer (5 mM Tris/HCl, 50 mM NaCl, pH = 7.39) and its concentration (4.325 mM) was spectrophotometrically determined using molar extinction coefficient 6600 M^-1^cm^-1^ at 260 nm. A 150 μM dilution in Tris/HCl buffer was prepared and used for the experiments [17]. A 2.0 mM stock solution of PND was freshly prepared in MQ water prior to use. The appropriate volume of this solution was added to 3 ml working solutions of 150 μM CT DNA to achieve molar ratios of 0.03, 0.06, 0.2 and 0.3 PND/DNA. Samples were prepared in triplicate and incubated for 30 minutes and 20 hours. All CD spectra of DNA and DNA/PND were recorded at 25°C over the range 205–380 nm and finally corrected with a blank and noise reduction. The final data is the average of three experiments and it is expressed in millidegrees (mdegs).

### Statistical analysis

For each data point, the average of triplicate and their corresponding standard deviations are reported. Statistical significance was determined through two-tailed paired Student’s *t*-tests, and a *P* value of <0.05 was deemed significant.

## Results

### PND exhibits selective cytotoxicity towards cancer cell lines

**Fig 2:**
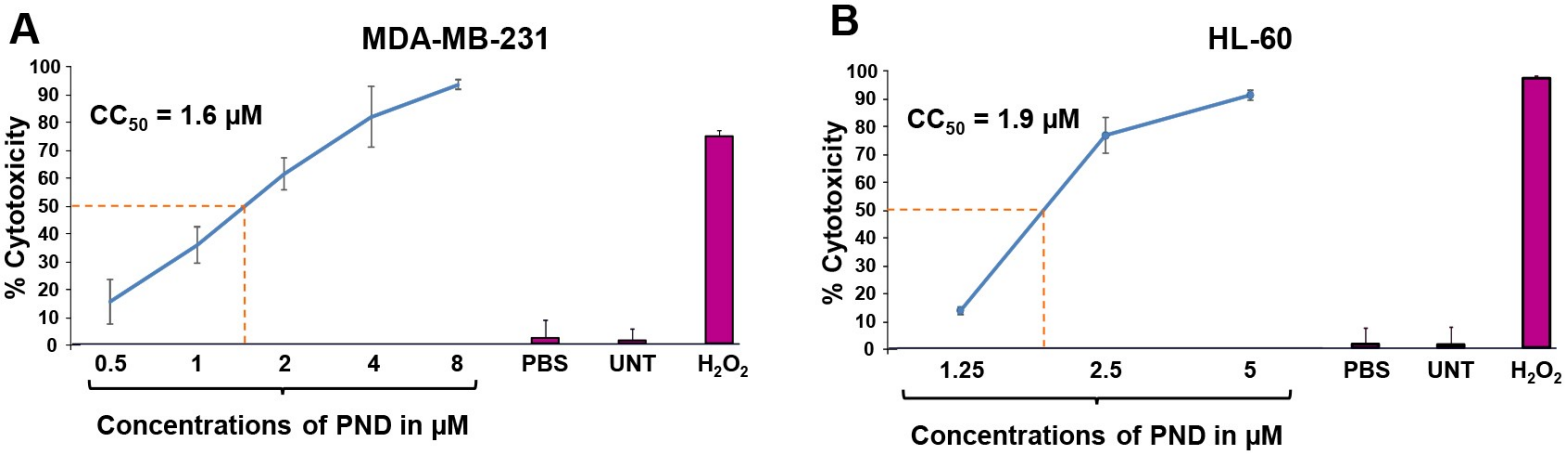
Representative PND dose-response curves utilized to determine the CC_50_ values. For these analyses, cells were exposed for 72 h to PND and cell viability was determined *via* the DNS assay. As an example, MDA-MB-231 (**A**) and HL-60 (**B**) were treated with a PND concentration gradient, as indicated on the x-axis while the percentage of cytotoxicity (dead cells) is shown on the y-axis. In this series of experiments, several controls were included: untreated cells and cells treated with the PBS diluent alone (0.5% v/v) were used as negative controls while 1 mM H_2_O_2_ was used as a positive control of cytotoxicity. Each experimental point represents the mean of four replicas and error bars their corresponding standard deviation. Cytotoxic concentration 50% (CC_50_) in micromolar (μM) units is defined as the concentration of PND required to perturb the plasma membrane of 50% of the cells after 72 h of incubation.

The potential cytotoxic effects PND were analyzed *via* a live cell imaging using the differential nuclear staining (DNS) assay [7], on seventeen human cancer cell lines and two non-cancerous control cell lines (MCF-10A and Hs-27; Table 1). For each individual cell line, dose-response curves were created, also using the DNS assay, to determine the CC_50_ of PND on these cell lines. In general, PND exerted a potent cytotoxicity on all cells tested with consistent CC_50_ values at low micromolar concentrations that ranged from 1.6 μM to 9.4 μM (Table 1). As shown in Fig 2A and 2B, the effects on various concentrations of PND on the MDA-MB-231 triple negative breast cancer cell line and the HL-60 acute promyelocytic leukemia cell line revealed that PND had CC_50_ values of 1.6 μM and 1.9 μM, respectively (Fig 2A and 2B). In these and other assays, untreated cells were included to determine if cell death occurred in the absence of treatment due to manual manipulation and/or incubation period. Solvent treated cells were also used as a control for non-specific cell death and for normalization purposes and H_2_O_2_-treated cells were used as positive controls for cytotoxicity (Fig 2). PND exerted a significant selective cytotoxicity (SCI) index on four out of six of breast cancer cell lines tested, MDA-MB-231, MDA-MB-231 LM2, MDA-MB-468 and MCF-7 with SCI values of 4.13, 2.54, 3.88 and 4.13, respectively, as compared with its non-cancerous breast MCF-10A cells (Table 1). Interestingly, the SCI values (<1) were not favorable on T47D and HCC-70 with values below 1 (Table 1). In addition, the highest SCI value on the leukemia/lymphoma cells tested corresponded to the HL-60 cell line with an SCI value of 3.5 (Table 1). Additionally, PND exhibited an SCI value of 3 and 3.3, for Ramos and Jurkat cells, respectively but poor selectivity was noticed for the CEM cell line (Table 1). Good selectivity was detected on the melanoma cell line (SCI = 3.2) and two of the three ovarian cancer lines tested (SCI = 3.9). However poor selectivity (<2.0) was detected on the pancreatic and lung cancer lines (Table 1). Since PND exhibited low CC_50_ values and showed significant selectivity (SCI >3) on both MDA-MB-231 and HL-60 cell lines, they were both selected for further analyses. Furthermore, since there are more limited therapies to triple-negative breast cancers (TNBCs), we have focused our characterization on the TNBC MDA-MB-231 cell line with the hope of adding another possible treatment to these types of cancers.

**Table 1:** PND cytotoxic concentration 50% (CC50) and selective cytotoxic index (SCI) on a panel of human cell lines.

Cell Type	Cell Line	CC_50_ μM	SCI
**Breast Cancer**	**MDA-MB-231**	**1.6 ± 0.20**†	**4.1**
**Breast Cancer**	**MDA-MB-231-LM2**‡	**2.6 ± 0.18**	**2.5**
**Breast Cancer**	**MDA-MB-468**	**1.7 ± 0.60**	**3.9**
**Breast Cancer**	**T47D**	**9.4 ± 1.41**	**0.7**
**Breast Cancer**	**HCC-1419**	**7.0 ± 0.33**	**0.9**
**Breast Cancer**	**MCF-7**	**1.6 ± 0.40**	**4.1**
**Breast Cancer**	**HCC-70**	**1.5 ± 0.51**	**4.4**
**Breast Epithelial**	**MCF10A**	**6.6 ± 0.42**	**1.0**
**Normal Fibroblast**	**HS-27**	**3.1 ± 0.39**	**2.1**
**Leukemia**	**HL-60**	**1.9 ± 0.07**	**3.5**
**Burkitt’s Lymphoma**	**Ramos**	**2.2 ± 0.16**	**3.0**
**T-cell Lymphoma**	**Jurkat**	**2.0 ± 0.11**	**3.3**
**T-cell Lymphoma**	**CEM**	**4.6 ± 0.09**	**1.4**
**Pancreatic Cancer**	**Panc-1**	**6.5 ± 0.18**	**1.0**
**Ovarian Cancer**	**Ovcar 8**	**1.7 ± 0.22**	**3.9**
**Ovarian Cancer**	**Ovcar 5**	**1.7 ± 0.06**	**3.9**
**Ovarian Cancer**	**Ovcar 3**	**3.3 ± 0.02**	**2**
**Lung Cancer**	**A549**	**3.5 ± 0.21**	**1.9**
**Melanoma**	**A375**	**2.0 ± 0.03**	**3.2**

^†^ Mean

± Standard Deviation

^‡^ Lung Metastatic (LM) variant of MDA- MB-231

### PND elicits phosphatidylserine externalization on both MDA-MB-231 and HL-60 cells

**Fig 3:**
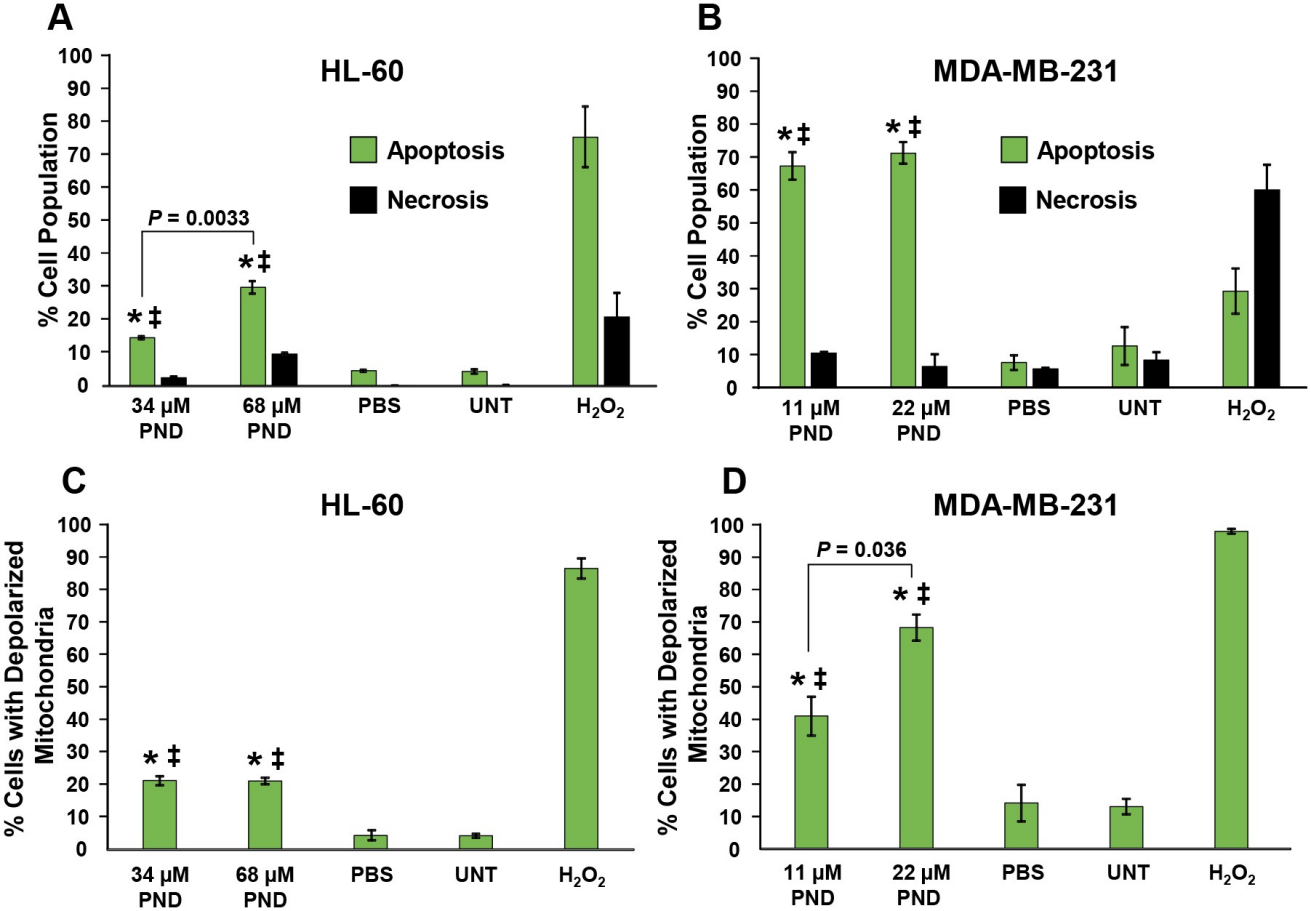
PND induced phosphatidylserine externalization on MDA-MB-231 (A) and HL-60 (B) cancer cells after 24 h of incubation. The cell death mechanism was studied after double staining of cells with annexin V-FITC and PI and monitored *via* flow cytometry. (**A-B**) The total percentages of cell undergoing apoptosis (*y*-axis) are expressed as the sum of both early and late stages of apoptosis (green bars); whereas cells stained only with PI and annexin V-FITC negative, were counted as the necrotic cell population (black bars). Calculations of two-tailed Student’s paired *t*-test of PND-treated cells as compared with PBS-treated (*) and untreated (‡) cells controls, provided consistently values of *P* < 0.001, in both circumstances. Each bar represents the mean of triplicates, and error bars the standard deviation of the mean. PND inflicted its cytotoxic effect *via* mitochondrial membrane depolarization on MDA-MB-231 **(C)** and HL-60 **(D)** cells. Cells were treated with PND for 6 h and changes in the mitochondrial membrane potential (*ΔΨm*) were monitored by staining them with JC-1 and examined *via* flow cytometry. The JC-1 reagent emits a green fluorescence signal after mitochondrial depolarization. (**C-D**) Percentages of cells emitting a green fluorescence signal, *y*-axis, versus different treatments, *x*-axis, are depicted. 1 mM of H_2_O_2_ was used as a positive control as it strongly perturbs mitochondrial membrane potential (*ΔΨm*). Each bar represents the mean of three replicates and error bar the standard deviation. Two-tailed Student’s paired *t*-test of PND-treated cells, as compared with PBS-treated (*) and untreated (‡) cell controls, provided consistent values of *P* < 0.01 and *P* < 0.001, respectively.

Phosphatidylserine (PS) externalization is an early feature of apoptosis induction that can be readily detected with the high-affinity PS ligand annexin V–FITC *via* flow cytometry [10,11,12]. To discern if PND induces its cytotoxicity through apoptosis or necrosis, cells were treated with two different concentrations of PND for 24 h, 34 μM and 68 μM for HL-60 and 11 μM and 22 μM for MDA-MB-231. Subsequently, cells were stained with annexin V-FITC and PI and analyzed *via* flow cytometry. PND was found to induce significant PS externalization in both cell lines as compared with positive and negative controls (*P*<0.001; Fig 3A and 3B). PND induced significant PS externalization in HL-60 cells in a dose-dependent manner, showing 14.8% and 30.2% of apoptotic cells at 34 μM and 68 μM, respectively (*P* = 0.0033, Fig 3A). Additionally, PND induced a higher percentage of PS externalization in MDA-MB-231 cells than HL-60 cells at both concentrations tested with 67.2% and 71.1% annexin positive cells at 11 μM and 22 μM, respectively (Fig 3B). As expected, solvent treated and untreated cells did not exhibit any significant increment in apoptotic or necrotic death (Fig 3A and 3B). Furthermore, H_2_O_2_ induced its cytotoxic effect *via* apoptosis and necrosis on MDA-MB-231 and HL-60 cells, respectively (Fig 3A and 3B). Thus PND induced PS externalization in both MDA-MB-231 and HL-60 cells, which is a well-known early event in the activation of apoptosis.

### PND induces mitochondrial depolarization on cancer cells

An early biochemical event triggering the intrinsic apoptosis pathway is mitochondrial depolarization, which can be quantified by using a polychromatic JC-1 reagent and flow cytometry [18,19,20]. JC-1 emits a red or green fluorescence signal when the mitochondria are polarized or depolarized, respectively. Consequently, both MDA-MB-231 and HL-60 cells were incubated for 6 h with PND and the mitochondrial membrane potential (*ΔΨm*) status was recorded. As expected based on the PS externalization data, both PND-treated cancer cells revealed significant mitochondrial depolarization, as compared to untreated and solvent treated cells (Fig 3C and 3D). These results indicate that PND is able to indue mitochondrial depolarization in both cancer cell types further indicating that PND induces cell death *via* the intrinsic apoptosis pathway.

### PND disrupts the cell cycle profile and displays DNA fragmentation on MDA-MB-231 and HL-60 cells

**Fig 4:**
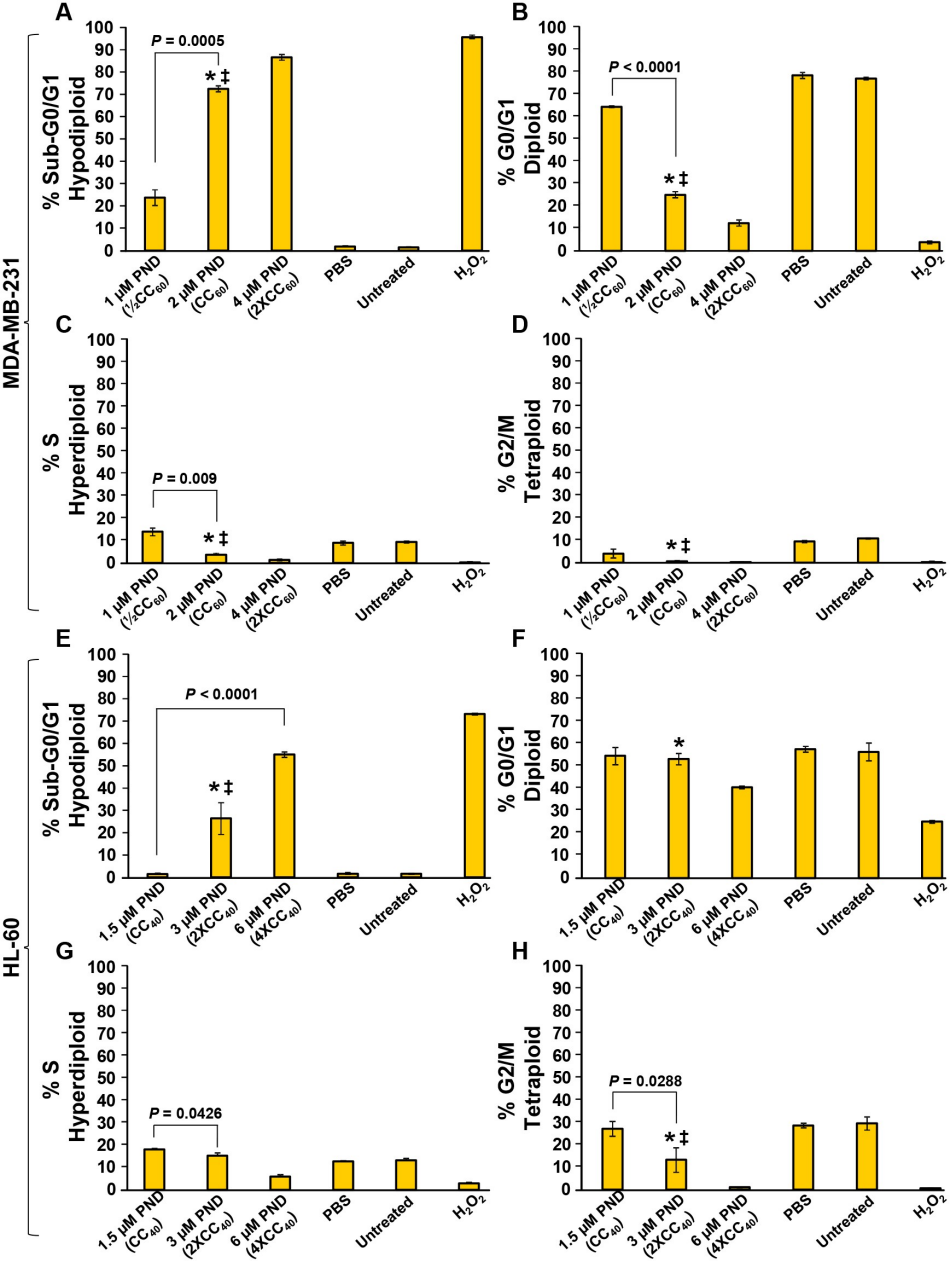
PND disturbed the cell-cycle profile of two cancer cell lines, MDA-MB-231 (A-D) and HL-60 (E-H), and also exhibited apoptosis-induced DNA fragmentation in a dose-dependent mode. After 72 h of PND treatment, cells were harvested, fixed, permeabilized, stained with DAPI and analyzed *via* flow cytometry. The percentages for each cell cycle phase are presented along with the y-axis, whereas the different treatments are displayed along the x-axis. For this series of experiments, the following controls were involved: untreated cells and cells treated with 0.1% PBS solvent were used as negative controls while 1 mM of H_2_O_2_ was used a positive control. Each bar denotes an average of three replicates, and the error bars indicate their corresponding standard deviation. For assay data acquisition and analysis purposes, the FL 9 detector, a single-cell gate and Kaluza flow cytometry software (Beckman Coulter) were utilized.

To examine if PND can affect the proliferation/cell division of MDA-MB-231 and HL-60 cells, the cell cycle distribution profile was examined *via* flow cytometry (Fig 4). To determine the effects of PND on cell cycle progression, a strategy to quantify cellular DNA content that depends on a violet-excited DNA intercalating fluorophore, DAPI (4',6-diamidino-2-phenylindole) was utilized [10,13]. After treatment of MDA-MB-231 cells with PND, a significant decrease of the G0/G1 cell subpopulation was observed; however this effect was only observed at the highest concentration of PND in HL-60 cells (Fig 4B and 4F). PND did not significantly affect or decreased the S and G2/M subpopulations in both MDA-MB-231 and HL-60 cells as compared with PBS and untreated controls (Fig 4B–4D and 4F–4H). Additionally, PND caused significant DNA fragmentation in a concentration-dependent fashion in both cancer cell lines, as denoted by a significant increase in the sub-G0/G1 subpopulation (*P*<0.0005; Fig 4A and 4E). Differences in the percentages of both cells in each phase of the cell cycle between PBS-treated and untreated cells were essentially indiscernible. Although the previous data were acquired from unsynchronized cell populations, results obtained with synchronized MDA-MB-231 and HL-60 cancer cell lines treated with PND (as in Fig 4) revealed few if any differences in the way the cells reacted to PND (see S1 Fig). As in Fig 4, PND elicited a dose-dependent increase in DNA fragmentation as shown by an increase in the sub-G0/G1 population in both cell lines (S1 Fig). As can be seen in S1 Fig, the results were almost identical to those observed with asynchronous populations of HL-60 and slightly depressed (lower) for MDA-MB-231. Cell cycle analyses were also performed on the untransformed breast epithelial cell line, MCF-10A, at high PND concentrations (matching the CC_50_ concentrations on MCF-10A; S2A Fig) and at lower concentrations. At high PND concentrations, a dose-dependent increase of sub G1/G0 was detected reflecting an increase in DNA degradation as expected from cells undergoing cell death (S2A Fig). However, at low PND concentrations (matching CC_50_ values on MDA-MD-231 as used in Fig 4; S2A Fig), little if any changes in DNA fragmentation were detected in the MCF-10A cell line. These experiments revealed that PND disrupted the distribution of the cell cycle and induced DNA fragmentation (sub-G0/G1 population) in both cancer cell types.

### PND interacts directly with double-stranded (ds) DNA

**Fig 5:**
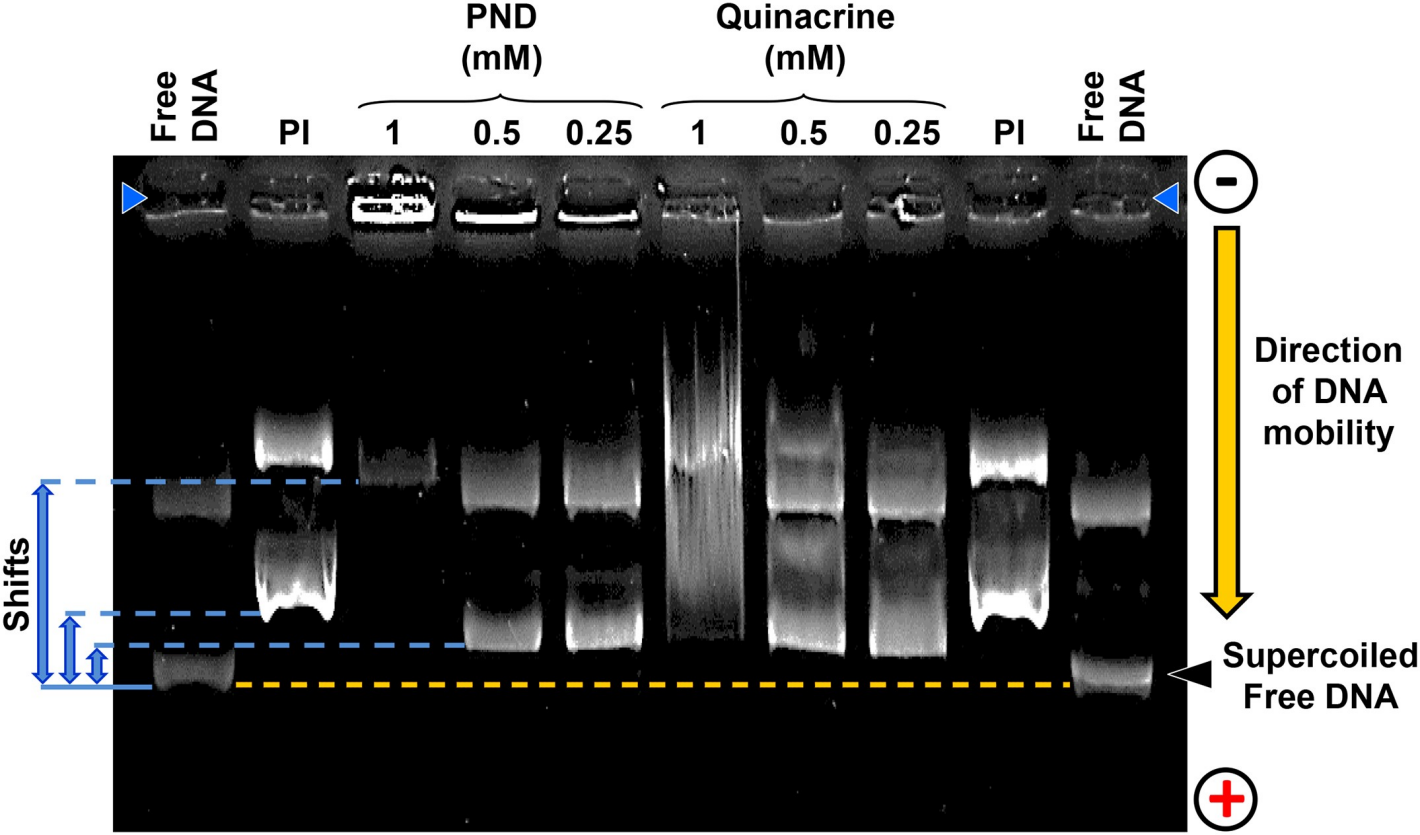
PND provoked DNA migration retardation in a dose-dependent manner. Three different concentrations of PND and quinacrine were incubated individually with a 100 ng of plasmid DNA and the potential of complex formation was analyzed *via* agarose gel electrophoresis. Reaction products were separated by 1% agarose gel electrophoresis in Tris/acetate/EDTA buffer and stained with Ethidium bromide. Both PI and free plasmid DNA were used as positive and negative controls of DNA mobility, respectively. The loading wells are located on the top of the image indicated by two blue head arrows (top left and right corners). The yellow dashed line is indicating the maximum mobility of the free supercoiled DNA is included as a reference. Three DNA mobility-shifts are indicated by blue lines and arrows (left side of the image). The migration direction of DNA is indicated by an arrow (right side of the image); from the cathode (negative) to the anode (positive). A representative image used to review the potential formation of DNA complexes is depicted.

The potential interaction between PND and dsDNA was examined with the use of a DNA mobility-shift assay using plasmid DNA as a binding substrate, and compared to that of quinacrine, a compound with comparable structure, and a well-known DNA intercalator. When 1 mM of PND was incubated with DNA a marked reduction of migration of PND-treated DNA was observed, as compared with free plasmid DNA (shifts are indicated to the left side of Fig 5). Approximately half of the total input DNA was located in the loading well with minimum mobility into the agarose gel. Furthermore, when 0.5 and 0.25 mM of PND were incubated with DNA, there was a clear reduction in mobility (noted to the left side of Fig 5). Additionally, PND-treatment did not result in DNA degradation based on the absence of DNA fragments smaller than the free supercoiled DNA (Fig 5) [14]. In previous studies, quinacrine, with a similar chemical structure to PND, was also found to interact with DNA by intercalation [21,22]. As shown in Fig 5, quinacrine caused the maximum retardation of DNA mobility at the highest concentration tested (1 mM) as evidenced by a smear, indicative of complexes with supercoiled DNA. Also, quinacrine provoked a clear retardation mobility of the DNA, similar than PND, when tested at 0.5 and 0.25 mM (Fig 5). As was the case with DNA treated with PND, quinacrine did not exhibit any DNA degradation activity (Fig 5). PI, which was used a positive control for DNA binding, also provoked retardation of DNA mobility. Our results, clearly indicate that PND can interact directly with DNA provoking its mobility-shift in agarose gels, and the similar behavior to that of quinacrine might suggest that PND also has the ability to intercalate between the bases of dsDNA.

### PND intercalates with DNA as determined by UV-Visible spectrophotometric titration

**Fig 6:**
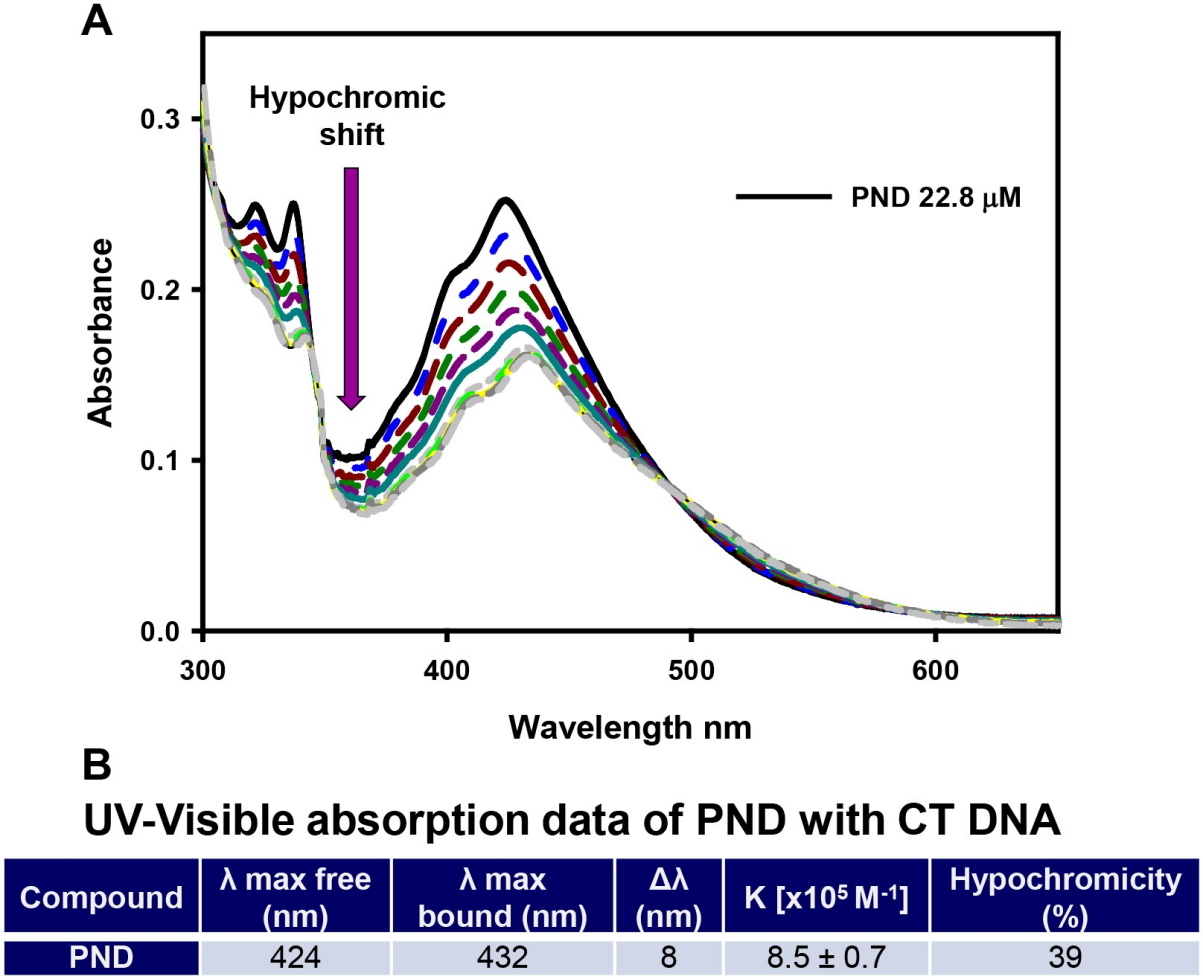
Calf Thymus DNA caused a hypochromic and batochromic effect on PND maxima of absorbance. UV-Visible spectrophotometric titration (300–650 nm) of PND (22.8 μM) in Tris/HCl buffer upon consecutive additions of Calf Thymus (CT) DNA (10.1 mM). The arrow indicates the spectral changes when DNA is added. (**B**) Summary of the UV-Visible titration data from PND and CT DNA interaction.

In order to further prove the intercalative interaction of PND with calf-thymus (CT) DNA, spectrophotometric titrations were performed in Tris/HCl buffer. PND displays strong absorption bands in the 300–500 nm region typical for transitions between electron energy levels of conjugated aromatic rings. In general, hypochromic and batochromic effects observed on maxima of UV-Visible absorbance can be taken as evidence of stacking interactions between conjugated aromatic systems that intercalate the nucleobases of DNA. Fig 6A shows the absorption spectra of PND in the studied region upon consecutive additions of CT DNA. The maximum of absorbance at 424 nm was studied to diagnose the compound-DNA interaction. Our results show a significant hypochromic effect (39%) and a red-shift of 8 nm. In addition, the Scatchard equation [15,16] was used to determine the binding affinity [8.5 ± 0.7 x 10^5^ M^-1^] of PND to CT DNA. All binding data of PND with CT DNA, shown in Fig 6B, are comparable to that of well-known intercalating agents with similar structure [23,24], and indicate that PND is also able to intercalate between the bases of DNA, as suggested by the mobility-shift assay.

### PND stabilizes the B conformation of CT DNA as observed by circular dichroism spectroscopy

**Fig 7:**
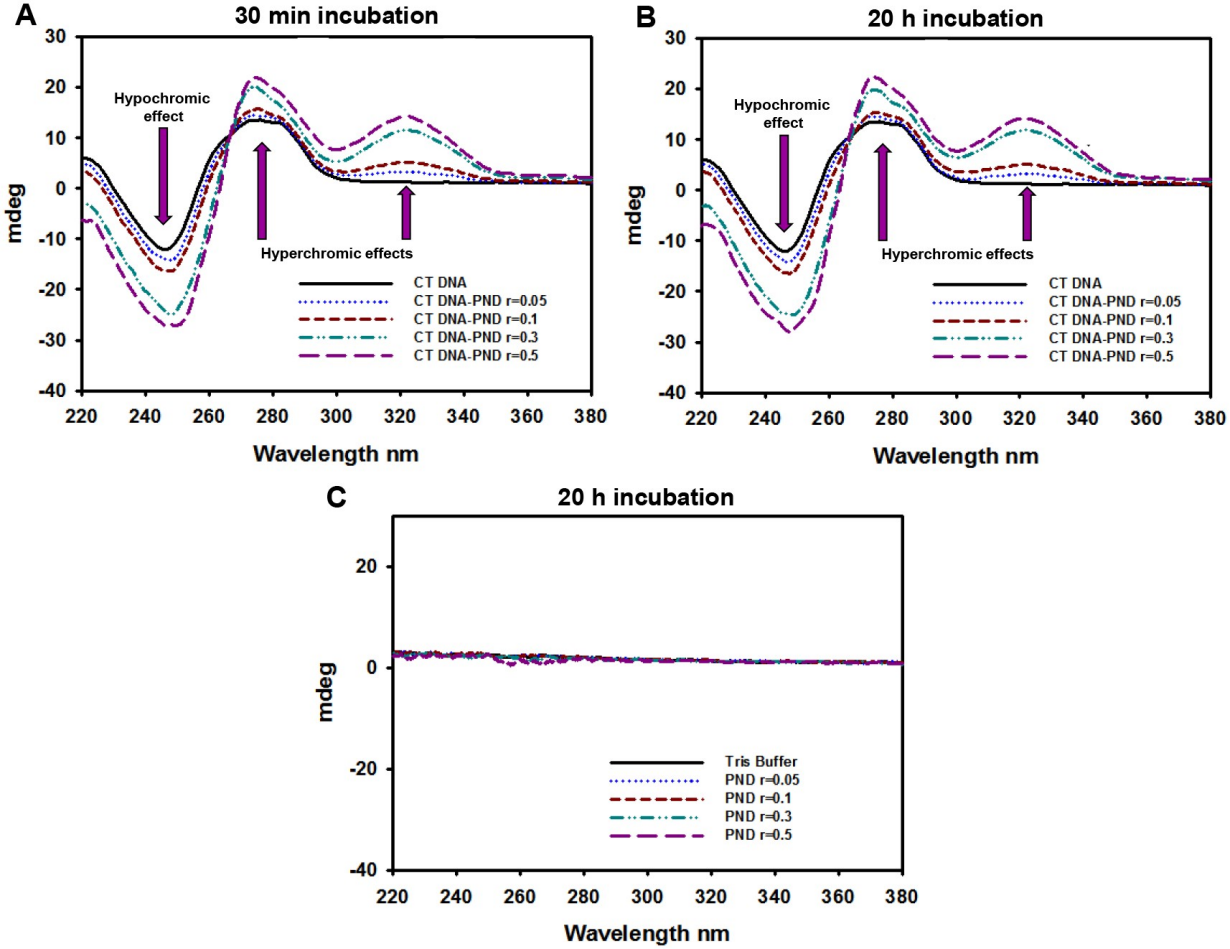
PND induced an increase in the intensity of the positive and negative bands of the circular dichroism spectra of Calf Thymus DNA. Changes in the circular dichroism (CD) spectra of Calf Thymus (CT) DNA as it interacts with PND. CT DNA (150 μM) in Tris/HCl buffer was subjected to CD analysis after 30 min (**A**) or 20 h (**B**) of incubation with PND at molar ratios of 0.03, 0.06, 0.2 and 0.3. The arrows specify the CD spectral changes of CT DNA under a gradient of increasing PND concentrations. Blanks of CD spectra with the same gradient of PND concentrations in absence of CT DNA incubated for 20 h (**C**). Millidegrees = mdegs.

In addition, detailed DNA conformational alterations were studied by means of circular dichroism spectroscopy in Tris/HCl buffer. A typical CD spectrum of CT DNA in its B form shows a positive band with a maximum at 275 nm due to base stacking, and a negative band with a minimum at 248 nm due to right-handed helicity [25]. Therefore, changes in the CD signals can be assigned to corresponding changes in DNA secondary structure. Fig 7A and 7B shows the CD spectrum of CT DNA, and the effect of treating it with increasing amounts of PND for 30 minutes and 20 hours, respectively. Our results show that PND was able to increase the intensity of both, the negative and the positive bands, in a concentration dependent manner but with no significant red-shifts in any of them. These results are comparable to previously reported similar compounds, and suggest that PND is able to stabilize the right-handed B form of DNA with no significant conformational changes [24,26]. The fact that the same spectral changes were observed upon 30 minutes and 20 hour incubations suggests that the kind of interaction taking place occurs in a few minutes, confirming the intercalative mode of binding. In addition, a positive signal appeared in the range 300–340 nm, a region where DNA does not absorb light, suggesting that some asymmetrical change is possibly being induced on PND upon binding to CT DNA [27], since no signal for PND is observed in absence of the nucleic acid (see Fig 7C).

## Discussion

In prior studies, PND was reported to significantly increase the sensitivity of multidrug-resistant K562/A02 and MCF-7/ADR cells to doxorubicin, while having no effect on the original K562 and MCF-7 cells [4]. In a subsequent report, gold nanorod complexes containing PND and DOX were found to be more effective than free DOX in their toxicity against MCF-7/ADR cancer cells and this toxicity was further enhanced by near-infrared radiation [6]. However, PND was not tested as a single agent in either report, nor was the mechanism of action determined, which provided the rationale fo the present work. It is also important to note that the MCF-7/ADR cells used in those studies are not derived from the original MCF-7 breast cancer cell line but instead were subsequently determined to be derived from the NCI/ADR-RES ovarian cancer cell line a derivative of OVCAR-8 [28].

In all the cancer cell lines analyzed, PND treatment was found to cause cell death at low micromolar concentrations (from 1.6 μM to 9.4 μM). Apart from being less toxic for non-cancerous cells (MCF-10A; CC_50_ of 6.6 μM), PND displayed favorable selectivity on breast, ovarian, and melanoma cancer cell lines, as compared with non-cancerous cells, with an SCI value greater than 2.5 (see Table 1). PND also exhibited favorable SCI values (from 1.43 to 3.47) on leukemia/lymphoma cells showing the highest selectivity on HL-60 cancer cells. The mode of action of PND was analyzed in both the MDA-MB-231 and HL-60 cancer cell lines. The triple-negative breast cancer (TNBC) MDA-MB-231 cells, which are highly aggressive, invasive, poorly differentiated, and lack the expression of estrogen receptors (ER), progesterone receptors (PR) and human epidermal growth factor receptor 2 (HER2) receptor; and due to these characteristics, these cells are widely used as a prototype of TBNC breast cancer [29]. The HL-60 cells are considered as a model system for studying human myeloid cell differentiation [30]. Both of these cancer cell lines were initially isolated from female patients and were used in our studies to avoid gender-related influences [31,32].

After exposure to a toxic agent, cells can undergo two main routes of cell death, apoptosis or necrosis [8]. Phosphatidylserine (PS) is preferentially located on the inner side of the plasma membrane leaflet, facing the cytosol, and when cells initiate the apoptosis pathway, it is translocated to the outer leaflet of the plasma membrane, which is a biochemical hallmark of apoptosis. Flow cytometry identified this apoptotic facet when using PI and FITC-conjugated annexin V which has high affinity for PS [11,12]. PND-treated MDA-MB-231 and HL-60 cells consistently exhibited PS externalization, suggesting that PND uses the apoptosis pathway to inflict its cytotoxicity in a dose-dependent manner.

Furthermore, apoptosis can be initiated *via* intrinsic or extrinsic biochemical pathways [33]. A critical biochemical event triggering the activation of the intrinsic pathway is through mitochondrial depolarization [34]. Therefore, MDA-MB-231 and HL-60 cells were exposed to PND and stained with the polychromatic JC-1 reagent, to investigate whether the mitochondrial depolarization was involved in its mechanism to induce cell death. Our data clearly indicate that both cancer cell lines exhibited a significant mitochondrial depolarization after exposure to PND, indicating that the intrinsic apoptosis pathway is induced.

A well-established strategy to study the cell cycle profile relies on quantifying the cellular DNA content *via* flow cytometry [10,13]. When using this approach, it is possible to distinguish three phases of the cell cycle, the G0/G1, S, and G2/M. In addition, independently of which initiation signal is used to activate the apoptotic cascade, the death signal ultimately results in DNA fragmentation; a late biochemical hallmark of apoptosis. When performing the cell cycle analysis *via* flow cytometry, cells undergoing DNA fragmentation are easy to identify by the presence of a sub-G0/G1 subpopulation, a distinct peak localized to the left side of the cell cycle histograms. Thus, the occurrence of apoptosis-induced DNA fragmentation as well as the cell cycle was analyzed concomitantly, after incubating the cells with PND for 72 h [10]. These assays revealed that PND was able to disturb the progression of the cell cycle in MDA-MB-231 and HL-60 cells, detecting a dissimilar pattern on each cell line. In addition, PND caused consistent DNA fragmentation in both MDA-MB-231 and HL-60 cells in a dose-dependent fashion, confirming the previous results that PND induces apoptosis.

The interaction between DNA and drugs or proteins can be readily detected by the retardation of DNA migration during the mobility-shift assay via gel electrophoresis [35]. In these DNA binding assays, the experimental compound-DNA complexes migrate more slowly than the free DNA (uncomplexed DNA control), which results in DNA having a heavier molecular weight. Additionally, when incubating a chemical compound with plasmid DNA, it is possible to detect if there are any deleterious effects resulting in DNA degradation or fragmentation [36]. Our results indicate that PND interacts directly with DNA since it causes significant DNA retardation in agarose gels, in a concentration-depended manner but did not cause DNA degradation [14]. Quinacrine was included in this series of experiments since it intercalates with dsDNA [37] and it exhibited a similar DNA retardation pattern as PND. Both PND and Quinacrine have a similar acridine backbone that provides a planar structure to both PND and quinacrine molecules, allowing them to intercalate by stacking into the base pairs of DNA (see Fig 1; [37]). The intercalative mode of DNA binding of PND was confirmed by UV-Visible spectrophotometric titrations as well as circular dichroism of CT DNA. Results from these DNA binding assays are comparable to those obtained with other DNA intercalating drugs [24,26,27] and, therefore, we hypothesize an analogous mode of interaction. In addition, CD spectra provided evidence of CT DNA stabilization in its B form with no detectable conformational changes upon interaction with PND. Collectively, the mobility-shift assay, UV-Visible and CD series of experiments, provide compeling evidence that PDN binds DNA by intercalating with nucleobases of the DNA.

In this preclinical study, our findings indicate that PND displays potent cytoxicity, with low micromolar CC_50_ values and favorable selective cytotoxicity index, towards a panel of human cancer cells, showing significant selectivity against triple negative breast cancer MDA-MB-231 cells (a class of aggressive tumors) and leukemia HL-60 cells. PND has potential as anti-breast and anti-leukemia cancer drug, since it markedly and consistently inflicts its cytotoxic effect by activating the apoptosis pathway, as evidenced by PS externalization, mitochondrial depolarization, DNA fragmentation, and interfering with the cell cycle. Furthermore, PND was found to interact directly with DNA by causing DNA retardation during agarose gel electrophoresis, and spectral changes in the UV-Visible profile of PND, as well as the CD spectra of DNA, suggesting an intercalative binding mode. To the best of our knowledge, this is the first report demonstrating that PND is capable of inducing apoptosis and capable of intercalating with dsDNA. Since PND has been used for decades in humans and an anti-malarial agent and it is not only effective but safe, this drug merits to be tested further as a repurposed drug for the treatment of cancer.

## Supporting information

S1 Fig Cell cycle analysis of synchronized MDA-MB-231 and HL-60 cell lines treated with PND. (DOCX)

S2 Fig Cell cycle analysis of MCF-10A cells treated with high and low concentrations of PND. (DOCX)
