# Supplementary material for: Pyronaridine exerts potent cytotoxicity on human breast and hematological cancer cells through induction of apoptosis
Source: PLoS One. 2018 Nov 5;13(11):e0206467. doi: 10.1371/journal.pone.0206467 (PMC6218039; doi:10.1371/journal.pone.0206467)
Supplement: S1 Fig — (DOCX) [file pone.0206467.s001.docx]

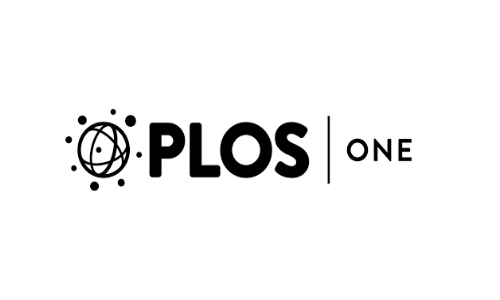


**Supporting Information**

**Pyronaridine exerts potent cytotoxicity on human breast and hematological cancer cells through induction of apoptosis**

**Paulina Villanueva, …. Renato J. Aguilera***

The Cytometry, Screening and Imaging Core Facility, Border Biomedical Research Center, Department of Biological Sciences, the University of Texas at El Paso, 500 West University Avenue, El Paso, Texas, USA 79968-0519.

*raguilera@utep.edu

**TABLE OF CONTENTS**

1. ***Supplementary Figure S1.* Cell Cycle Analysis of PND-Treated synchronized MDA-MB-231 and HL-60**
2. ***Supplementary Figure S2.*** **Cell Cycle Analysis of PND-Treated MCF-10A**

**CONTENTS**

1. ***Supplementary*** *Figure S1.* **Cell Cycle Analysis of PND-Treated synchronized MDA-MB-231 and HL-60**

***Figure S1.* PND disturbed the cell-cycle profile of synchronized cancer cell lines, MDA-MB-231 (A-D) and HL-60 (E-H), and also exhibited apoptosis-induced DNA fragmentation in a dose-dependent mode.** After 72 h of PND treatment, cells were harvested, fixed, permeabilized, stained with DAPI and analyzed *via* flow cytometry. The percentages for each cell cycle phase are presented along with the y-axis, whereas the different treatments are displayed along the x-axis. For this series of experiments, the following controls were involved: untreated cells and cells treated with 0.1% PBS solvent were used as negative controls while 1 mM of H_2_O_2_ was used a positive control. Each bar denotes an average of three replicates, and the error bars indicate their corresponding standard deviation. For assay data acquisition and analysis purposes, the FL 9 detector, a single-cell gate and Kaluza flow cytometry software (Beckman Coulter) were utilized.
