# Supplementary material for: Pyronaridine exerts potent cytotoxicity on human breast and hematological cancer cells through induction of apoptosis
Source: PLoS One. 2018 Nov 5;13(11):e0206467. doi: 10.1371/journal.pone.0206467 (PMC6218039; doi:10.1371/journal.pone.0206467)
Supplement: S2 Fig — (DOCX) [file pone.0206467.s002.docx]

1. ***Supplementary*** *Figure S2.* **Cell Cycle Analysis of PND-Treated MCF-10A cells at high and low concentrations**

***Figure S2.* PND disturbed the cell-cycle profile of a non-cancerous MCF-10A cell line at high concentrations (A), but did not exhibit apoptosis-induced DNA fragmentation and induced G0/G1 phase arrest at lower concentrations (B).** After 72 h of PND treatment, cells were harvested, fixed, permeabilized, stained with DAPI and analyzed *via* flow cytometry. The percentages for each cell cycle phase are presented along with the y-axis, whereas the different treatments are displayed along the x-axis. For this series of experiments, the following controls were involved: untreated cells and cells treated with 0.1% PBS solvent were used as negative controls while 1 mM of H_2_O_2_ was used a positive control. Each bar denotes an average of three replicates, and the error bars indicate their corresponding standard deviation. For assay data acquisition and analysis purposes, the FL 9 detector, a single-cell gate and Kaluza flow cytometry software (Beckman Coulter) were utilized.
